# Age, participation in competitive sports, bony lesions, ALPSA lesions, > 1 preoperative dislocations, surgical delay and ISIS score > 3 are risk factors for recurrence following arthroscopic Bankart repair: a systematic review and meta-analysis of 4584 shoulders

**DOI:** 10.1007/s00167-021-06704-7

**Published:** 2021-08-22

**Authors:** Lukas P. E. Verweij, Sanne H. van Spanning, Adriano Grillo, Gino M. M. J. Kerkhoffs, Simone Priester-Vink, Derek F. P. van Deurzen, Michel P. J. van den Bekerom

**Affiliations:** 1 Amsterdam UMC, Department of Orthopedic Surgery, University of Amsterdam, Amsterdam Movement Sciences, Location AMC, Meibergdreef 9, 1105 AZ Amsterdam, The Netherlands; 2 Academic Center for Evidence-Based Sports Medicine (ACES), Amsterdam, The Netherlands; 3 Amsterdam Collaboration on Health and Safety in Sports (ACHSS), AMC/VUmc IOC Research Center, Amsterdam, Netherlands; 4 Department of Orthopedic Surgery, Shoulder and Elbow Unit, OLVG, Amsterdam, The Netherlands; 5 Department of Research and Epidemiology, OLVG, Amsterdam, The Netherlands; 6 Faculty of Behavioural and Movement Sciences, Department of Human Movement Sciences, Vrije Universiteit Amsterdam, Amsterdam Movement Sciences, Amsterdam, the Netherlands

**Keywords:** Shoulder, Anterior dislocation, Instability, Risk, Bankart

## Abstract

**Purpose:**

Determining the risk of recurrent instability following an arthroscopic Bankart repair can be challenging, as numerous risk factors have been identified that might predispose recurrent instability. However, an overview with quantitative analysis of all available risk factors is lacking. Therefore, the aim of this systematic review is to identify risk factors that are associated with recurrence following an arthroscopic Bankart repair.

**Methods:**

Relevant studies were identified by searching PubMed, Embase/Ovid, Cochrane Database of Systematic Reviews/Wiley, Cochrane Central Register of Controlled Trials/Wiley, CINAHL/Ebsco, and Web of Science/Clarivate Analytics from inception up to November 12th 2020. Studies evaluating risk factors for recurrence following an arthroscopic Bankart repair with a minimal follow-up of 2 years were included.

**Results:**

Twenty-nine studies met the inclusion criteria and comprised a total of 4582 shoulders (4578 patients). Meta-analyses were feasible for 22 risk factors and demonstrated that age ≤ 20 years (RR = 2.02; *P* < 0.00001), age ≤ 30 years (RR = 2.62; *P* = 0.005), participation in competitive sports (RR = 2.40; *P* = 0.02), Hill-Sachs lesion (RR = 1.77; *P* = 0.0005), off-track Hill-Sachs lesion (RR = 3.24; *P* = 0.002), glenoid bone loss (RR = 2.38; *P* = 0.0001), ALPSA lesion (RR = 1.90; *P* = 0.03), > 1 preoperative dislocations (RR = 2.02; *P* = 0.03), > 6 months surgical delay (RR = 2.86; *P* < 0.0001), ISIS > 3 (RR = 3.28; *P* = 0.0007) and ISIS > 6 (RR = 4.88; *P* < 0.00001) were risk factors for recurrence. Male gender, an affected dominant arm, hyperlaxity, participation in contact and/or overhead sports, glenoid fracture, SLAP lesion with/without repair, rotator cuff tear, > 5 preoperative dislocations and using ≤ 2 anchors could not be confirmed as risk factors. In addition, no difference was observed between the age groups ≤ 20 and 21–30 years.

**Conclusion:**

Meta-analyses demonstrated that age ≤ 20 years, age ≤ 30 years, participation in competitive sports, Hill-Sachs lesion, off-track Hill-Sachs lesion, glenoid bone loss, ALPSA lesion, > 1 preoperative dislocations, > 6 months surgical delay from first-time dislocation to surgery, ISIS > 3 and ISIS > 6 were risk factors for recurrence following an arthroscopic Bankart repair. These factors can assist clinicians in giving a proper advice regarding treatment.

**Level of evidence:**

Level IV.

**Supplementary Information:**

The online version contains supplementary material available at 10.1007/s00167-021-06704-7.

## Introduction

Shoulder instability is characterized by dislocation or subluxation of the glenohumeral joint or a feeling of apprehension. The estimated incidence rate in the United States is 23.9 per 100,000 person-years and the cause is often traumatic [59]. Furthermore, over 95% of shoulder dislocations occur in the anterior direction, in contrast to the less frequently occurring posterior and inferior dislocations [50, 59]. Shoulder dislocations limit patients in activities of daily living and sports and are associated with development of osteoarthritis [51, 60]. In addition, recurrent instability includes high social costs and performing operative treatment following a first-time dislocations is demonstrated to be cost-effective [9, 47]. In a prospective study with 25 years of follow-up, Hovelius et al. demonstrated a recurrence rate of up to 60% following non-operative treatment, which generally consists of scapula and rotator cuff training [8, 19]. Operative treatment may have been beneficial for these patients.

The most commonly performed surgical treatment options include repair of the labrum with/without tenomyodesis of the infraspinatus tendon (remplissage) and bone augmentation of the glenoid [50]. Counseling patients for operative treatment can be challenging as the risks and benefits for each individual patient must be weighed. The arthroscopic labral repair demonstrates a recurrence rate of 16%, whereas the open bone augmentation procedures are more effective and show a recurrence rate of 2–6% [21, 55]. However, these procedures are more invasive and demonstrate a complication rate of 5–14% compared to < 2% following the arthroscopic repair [21, 55]. Numerous studies have investigated if glenoid bone loss has a cut-off value that can advise professionals for which cases to perform a bone augmentation procedure [42, 52]. However, an objective cut-off value that predicts recurrent instability has yet to be found and the current methods only measure bone loss in 2D, therefore not taking the 3D morphology into account [52]. Furthermore, other methods that might be able to determine recurrence risk are proposed. These include the instability severity index score (ISIS), glenoid morphology (i.e. concavity, version, inclination), an off-track Hill-Sachs lesion and translation of the humeral head [2, 11, 27, 29, 56]. These methods, or a combination of these methods, seem promising to objectively determine recurrence risk in the future. Currently, mainly risk factors based on group averages are used to predict recurrence risk following an arthroscopic Bankart repair. The most recent systematic review evaluating these factors was published almost a decade ago by Randelli et al. [38]. This review demonstrates that there is no consensus regarding which risk factors predispose recurrence and therefore demands a quantitative analysis. In addition, since then many studies evaluating new risk factors have been published that need to be included in the overview [10, 24, 30]. Therefore, the aim of this systematic review is to identify risk factors that are associated with recurrence following an arthroscopic Bankart repair.

## Materials and methods

This systematic review was carried out in accordance with the PRISMA (Preferred Reporting Items for Systematic Reviews and Meta-Analyses) protocol and registered with the PROSPERO database (registration number: CRD42020212423)[28].

### Literature search

Relevant studies were identified by searching PubMed, Embase/Ovid, Cochrane Database of Systematic Reviews/Wiley, Cochrane Central Register of Controlled Trials/Wiley, CINAHL/Ebsco, and Web of Science/Clarivate Analytics from inception up to November 12th 2020. The following terms, including synonyms and closely related words, were used as index terms or free-text words: ‘shoulder’, ‘dislocation’, ‘Bankart’ and ‘recurrence’ or ‘tear’. Full search strategies for all databases are available in supplementary 1. No language or other restrictions were applied to any of the searches. Duplicate articles were excluded by the information specialist using EndNote X8 (Clarivate analytics, Philadelphia, Pennsylvania, United States). Studies that met the inclusion criteria were screened full-text. In addition, the reference list of each study was assessed to find other possibly relevant studies. Both title/abstract screening and full-text screening were performed by two authors (L.P.E. V. and S.H. S.), with the use of Rayyan [32]. Any disagreement was resolved by discussion and consensus. If the authors were unable to reach a consensus, a third author (M.P.J. B.) would give final judgment.

### Inclusion and exclusion criteria

Prospective and retrospective cohort studies evaluating risk factors for recurrence following an arthroscopic Bankart repair with a minimum follow-up of 2 years were included. Recurrence was defined as a complete anterior shoulder dislocation or subluxation. Comparative studies were only included when independent risk factors were identified. Only articles written in the English, Dutch, German or Italian language were included. Studies were excluded if the mean age was less than 18 years or when the main focus was patients with posterior, multidirectional or atraumatic instability. In addition, reviews, cadaveric studies, software simulations, case reports, animal studies, abstracts, book chapters and studies evaluating recurrence following other treatments than an arthroscopic Bankart repair were excluded. When the same cohort of patients was used, the study with the longest follow-up was included.

### Quality appraisal

The methodological quality of the selected studies was assessed using the Methodological Index for Non-Randomized Studies (MINORS) tool [44]. The included comparative studies that determined independent risk factors were seen as non-comparative studies during the assessment. A non-randomized non-comparative study can earn a maximum of 16 points using the MINORS tool. The assessment was performed by two authors (L.P.E. V. and S.H. S.). Following the assessment, the authors compared the results to create a final rating for the individual studies. If the authors were unable to reach a consensus, a third author (M.P.J. B.) would give final judgment.

### Data extraction

Extracted baseline patient characteristics included sample size, gender, mean age at surgery and follow-up. The primary outcome was recurrence following an arthroscopic Bankart repair. An arthroscopic Bankart repair was defined as any form of arthroscopic anterior capsulolabral repair without tenomyodesis of the infraspinatus tendon. Data of risk factors were extracted if the proportion of recurrence for patients with and without a specific risk factor could be extracted or calculated. If proportions could not be extracted or calculated for any risk factor in studies published after 2010, the authors were sent an email and asked to share the data. Risk factors included age at surgery, gender, if the dominant arm was affected, hyperlaxity as defined by the authors, participation in contact and/or overhead sports, participation in competitive sports, Hill-Sachs lesions, if the Hill-Sachs lesion was off-track, glenoid bone loss, glenoid fractures, Anterior Labroligamentous Periosteal Sleeve Avulsion (ALPSA) lesions, Glenolabral Articular Disruption (GLAD) lesions, Superior Labral Anterior Posterior (SLAP) lesions, rotator cuff tears, time from first-time dislocation to surgical treatment, number of preoperative dislocations, Multiple Subscapularis Tendon Sign (MSTS) and ISIS. As variety in definition for specific risk factors was present, the definition of the original articles was adopted. Arthroscopy was considered the gold standard to identify any lesion. However, lesions identified with either radiographs, CT or MRI were extracted as well. If percentage of glenoid bone loss was measured, it was considered to be present when > 5% was measured with any glenoid bone loss measuring method using CT, MRI or during arthroscopy [10, 52]. Data were extracted to Excel (Microsoft Corporation. Microsoft Excel [Internet]. 2016. Available from: https://office.microsoft.com/excel).

### Statistical analysis

Patient characteristics and length of follow-up were pooled by calculation of weighted means and pooled standard deviations. If the standard deviation was not reported, it was estimated with the range and the sample size according to Walter et al. [54]. Furthermore, if the mean was not reported, it was estimated using the median, range and sample size according to Hozo et al. [20]. Proportion of recurrence was calculated for patient groups with and without a specific risk factor. If only an odds ratio was reported, the proportions were calculated when sufficient variables were available for the calculation. If possible, risk factor data were pooled to perform meta-analyses, including ≥ 2 studies. Since small differences were observed for the risk factor age at time of surgery (i.e. age < 20 and age ≤ 20 years), a deviation of one year was accepted to pool the data. Proportions were compared by use of Χ^2^ tests. Review Manager version 5.3 (the Nordic Cochrane Center, Copenhagen, Denmark) was used to perform meta-analyses and calculate risk ratios (RR) with 95% confidence interval (CI). Heterogeneity between studies was assessed by use of the *I*^2^ statistic[18].

## Results

### Screening and study characteristics

**Fig. 1:**
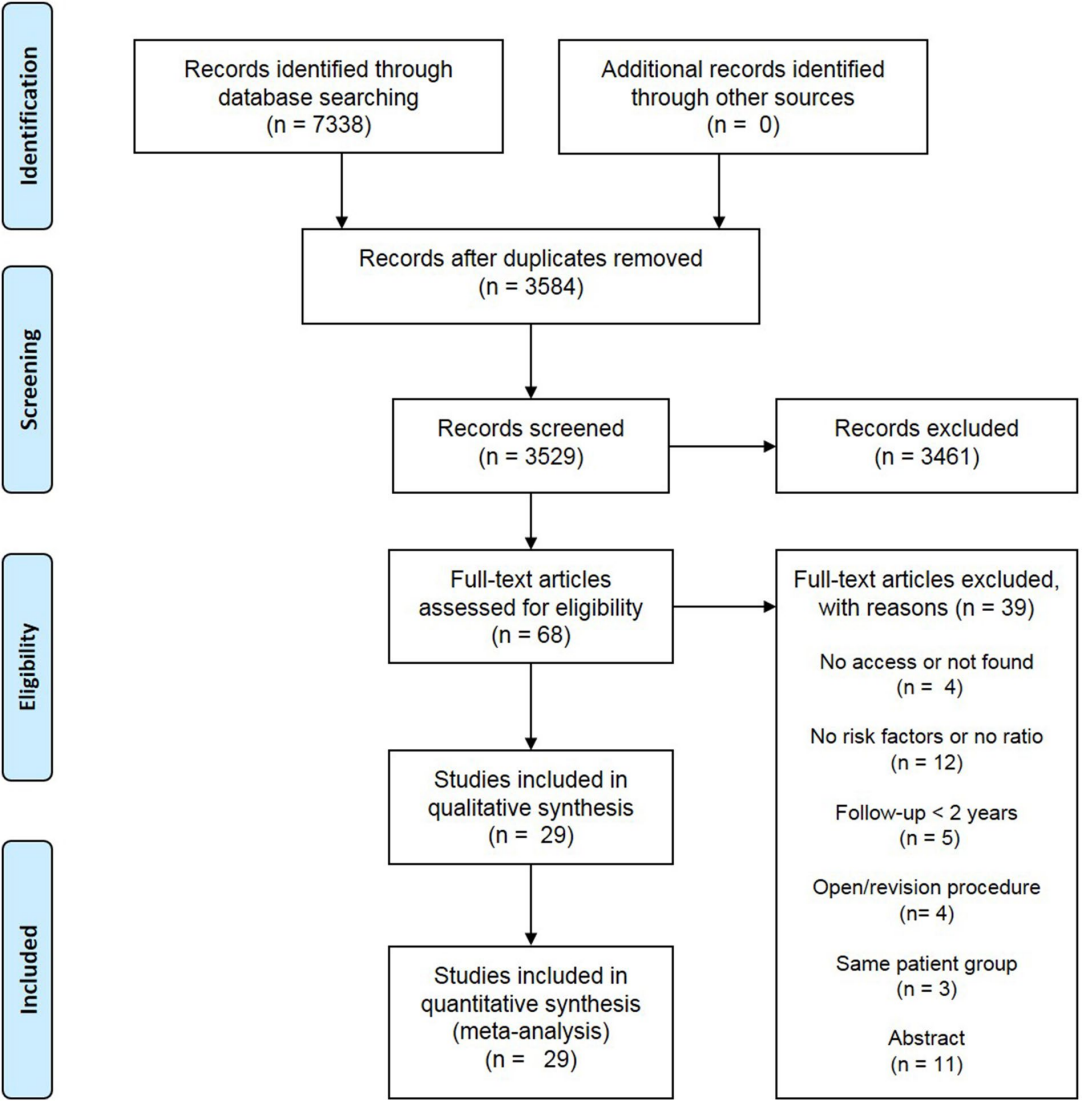
Flow diagram

After duplicates were removed, the titles and abstracts of 3584 studies were screened (Fig. 1). Sixty-seven studies were included in the full-text screening of which 28 studies met the inclusion criteria. Three study groups that published their manuscript after 2010 were asked to share their data as proportions could not be calculated. One research group replied, which created a total of 29 inclusions for analysis [46]. Reasons for exclusion during the full-text screening are listed in Fig. 1. Seven prospective [4, 5, 10, 37, 46, 48, 53] and 22 retrospective studies [1, 3, 6, 7, 14–17, 22–25, 30, 31, 33, 35, 36, 43, 45, 49, 57, 58] were included (Table 1). The included studies comprised a combined sample size of 4584 shoulders (range 51–670) in 4564 patients (supplementary 2). The weighted mean age at surgery was 27.2 years (range 10–67) and 82% of patients were male. The weighted mean follow-up was 6.3 years (range 2–14.3) and during this follow-up period a weighted recurrence rate of 17% (range 6%–35%) was observed. The MINORS ranged from 7 to 14 points (Table 1 & supplementary 3). In total, 22 risk factors could be quantitatively analyzed.

**Table 1:** Study characteristics

Author	Year	Design	Sample size	Male (%)	Mean age at operation (y)	Mean follow-up (y)	Recurrence (%)	MINORS
Hayashida et al. [17]	1998	R	82	77	21.0 ± 7.6	3.3 ± 0.8	18	7
Calvo et al. [5]	2005	P	61	85	27.5 ± 10.8	3.7 ± 1.4	18	12
Porcellini et al. [37]	2009	P	385	72	28.7 ± 8.2	3.0 ± 0.0	8	8
Flinkkilä et al. [14]	2010	R	174	72	28.0 ± 9.0	4.6 ± 1.1	19	8
Voos et al. [53]	2010	P	73	84	32.6 ± 8.4	2.8 ± 0.4	15	12
Van der Linde et al. [48]	2011	P	68	66	31.0 ± 7.8	9.0 ± 0.4	35	12
Sommaire et al. [45]	2012	R	77	79	27.5 ± 8.7	3.7 ± 0.3	16	7
Bessiere et al. [3]	2013	R	51	86	26.0 ± 6.9	5.3 ± 0.4	24	9
Bouliane et al. [4]	2014	P	100	77	25.2 ± 9.0	2.0 ± 0.0	6	14
Shibataet al. [43]	2014	R	102	81	25.7 ± 5.2	5.6 ± 1.6	9	10
Phadnis et al. [35]	2015	R	141	78	27.1 ± 8.9	3.9 ± 2.2	13	10
Gasparini et al. [15]	2016	P	143	90	25.0 ± 8.1	7.5 ± 2.3	23	8
Aboalata et al. [1]	2017	R	143	75	28.2 ± 8.3	13.3	18	7
Nakagawa et al. [30]	2017	R	113	88	18.3 ± 3.8	> 2	20	8
Pogorzelski et al. [36]	2018	R	62	84	21.5 ± 3.5	6.5 ± 3.5	16	7
Lee et al. [24]	2018	R	170	89	22.7 ± 3.0	3.1 ± 0.6	19	11
Yang et al. [57]	2018	R	160	90	27.7 ± 6.4	6.4 ± 2.0	14	10
Gul et al. [16]	2019	R	62	52	26.7 ± 8.0	2.4 ± 1.1	8	10
Chan et al. [6]	2019	R	131	91	26.8 ± 5.3	> 2	26	10
Loppini et al. [25]	2019	R	670	85	27.0 ± 3.5	8.8 ± 1.3	17	8
Iban et al. [22]	2019	R	142	83	35.5 ± 7.9	5.3 ± 1.2	14	9
Thomazeau et al. [46]	2019	P	125	68	30.2 ± 9.0	9.0 ± 0.0	19	10
Vermeulen et al. [49]	2019	R	147	76	30.0 ± 11.1	6.3 ± 1.7	22	8
Ono et al. [31]	2019	R	51	88	27.0 ± 7.3	10.1 ± 0.7	31	7
Kanatli et al. [23]	2019	R	87	86	28.4 ± 10.8	6.8 ± 2.3	10	9
Chen et al. [7]	2020	R	222	88	25.0 ± 7.8	4.2 ± 0.8	14	11
Panzram et al. [33]	2020	R	100	76	27.8 ± 1.2	8.3 ± 2.2	22	8
Yian et al. [58]	2020	R	337	83	–	6.2 ± 1.0	30	10
Dekker et al. [10]	2020	P	405	89	27.5 ± 4.8	5.1 ± 0.7	15	10

*R* retrospective, *P* prospective

### Patient factors

**Fig. 2:**
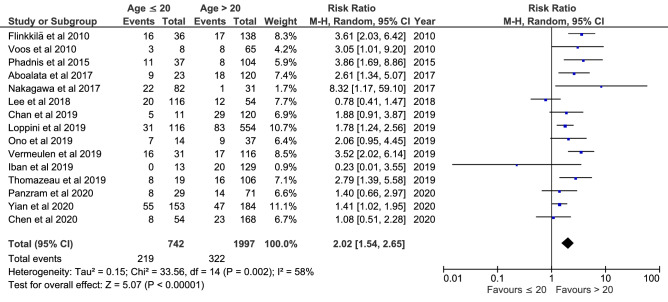
Meta-analysis of risk factor age ≤ 20 years

**Fig. 3:**
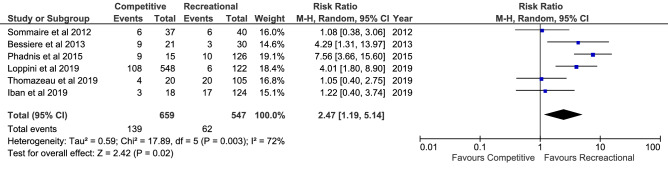
Meta-analysis of risk factor participation in competitive sports

**Fig. 4:**
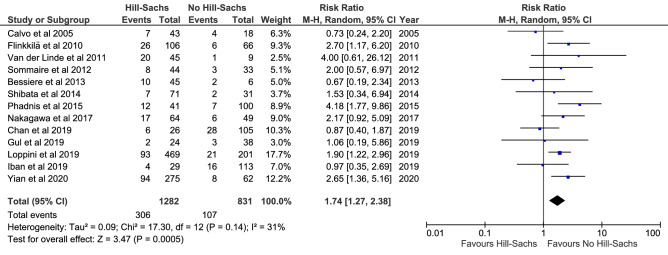
Meta-analysis of risk factor Hill-Sachs lesion

**Fig. 5:**
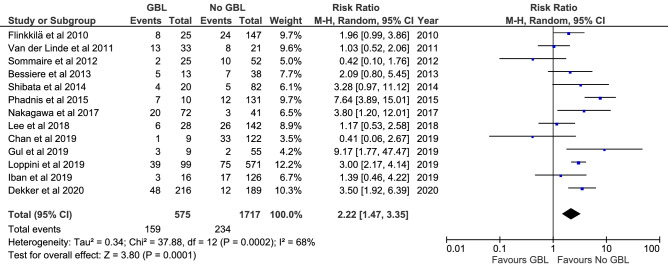
Meta-analysis of risk factor glenoid bone loss. GBL = glenoid bone loss

Meta-analyses were feasible for patient age, gender, an affected dominant arm and hyperlaxity. Fifteen studies (2739 shoulders) demonstrated a higher recurrence risk in patients aged ≤ 20 years compared to patients > 20 years (RR = 2.02; *P* < 0.00001; *I*^2^ = 58%; Fig. 2); five studies (588 shoulders) demonstrated a higher recurrence risk in patients aged ≤ 30 years compared to patients > 30 years (RR = 2.62; *P* = 0.005; *I*^2^ = 57%; supplementary 4, Fig. 1); seven studies (622 shoulders) demonstrated no difference in recurrence risk when comparing patients aged ≤ 20 years and 21–30 years (RR = 1.66; n.s.; *I*^2^ = 65%; supplementary 4, Fig. 2); 18 studies (2973 shoulders) demonstrated no difference in recurrence risk when comparing males and females (RR = 1.10; n.s.; *I*^2^ = 43%; supplementary 4, Fig. 3); seven studies (1008 shoulders) demonstrated no difference in recurrence risk when comparing patients where the dominant arm was affected and patients where the non-dominant arm was affected (RR = 0.76; n.s.; *I*^2^ = 0%; supplementary 4, Fig. 4); 10 studies (1670 shoulders) demonstrated no difference in recurrence risk when comparing patient with and without hyperlaxity (RR = 1.21; n.s.; *I*^2^ = 47%; supplementary 4, Fig. 5).

### Sports participation

**Fig. 6:**
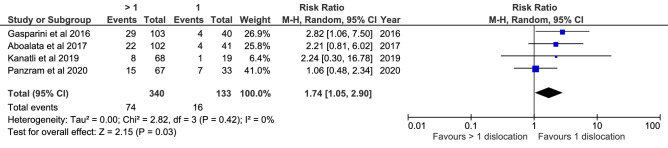
Meta-analysis of risk factor > 1 preoperative dislocations

Meta-analyses were feasible for participation in competitive, contact and overhead sports. Six studies (1,206 shoulders) demonstrated a higher recurrence risk in patients participating in competitive sports compared to recreational or no sports (RR = 2.47; *P* = 0.02; *I*^2^ = 72%; Fig. 3); 11 studies (1,746 shoulders) demonstrated no difference when comparing participation in contact or overhead sports with other or no sports (RR = 1.51; n.s.; *I*^2^ = 71%; supplementary 4, Fig. 6); seven studies (869 shoulders) demonstrated no difference when comparing participation in contact sports only with other or no sports (RR = 1.50; n.s.; *I*^2^ = 53%; supplementary 4, Figure  7); five studies (724 shoulders) demonstrated no difference when comparing participation in overhead sports only with other or no sports (RR = 0.64; n.s.; *I*^2^ = 44%; supplementary 4, Figure 8).

### Bony lesions or bone loss

Meta-analyses were feasible for presence of a Hill-Sachs lesion or glenoid bone loss, glenoid fracture and an off-track Hill-Sachs lesion. Fourteen studies (2113 shoulders) demonstrated a higher recurrence risk in patients with a Hill-Sachs lesion compared to patients where the lesions was not reported (RR = 1.74; *P* = 0.0005; *I*^2^ = 31%; Fig. 4); three studies (667 shoulders demonstrated a higher recurrence risk in patients with an off-track Hill-Sachs lesion compared to patients with an on-track lesion (RR = 3.24; *P* = 0.002; *I*^2^ = 84%; supplementary 4, Figure 9); 13 studies (2113 shoulders) demonstrated a higher recurrence risk in patients with a glenoid bone loss compared to patients without bone loss (RR = 2.22; *P* = 0.0001; *I*^2^ = 68%; Fig. 5); four studies (338 shoulders) demonstrated no difference when comparing patients with a glenoid fracture to patients where the fracture was not reported (RR = 1.01; n.s.; *I*^2^ = 34%; supplementary 4, Figure 10).

### Soft-tissue lesions

Meta-analyses were feasible for presence of an ALPSA, any SLAP lesion, SLAP lesion with repair and a rotator cuff lesion. Three studies (523 shoulders) demonstrated a higher recurrence risk in patients with an ALPSA lesion compared to patients where the lesions was not reported (RR = 1.90; *P* = 0.03; *I*^2^ = 0%; supplementary 4, Figure 11); five studies (610 shoulders) demonstrated no difference when comparing patients with any SLAP lesion compared to patients where the lesions was not reported (RR = 0.72; n.s.; *I*^2^ = 0%; supplementary 4, Figure 12); three studies (278 shoulders) demonstrated no difference when comparing patients with a SLAP lesion with repair compared to patients where the lesions was not reported and a repair was not indicated (RR = 0.58; n.s.; *I*^2^ = 0%; supplementary 4, Figure 13); two studies (344 shoulders) demonstrated no difference when comparing patients with a rotator cuff tear compared to patients where the tear was not reported (RR = 0.96; n.s.; *I*^2^ = 0%; supplementary 4, Figure 14). A meta-analysis for GLAD lesions was not feasible, however Pogorzelski et al. found a higher recurrence rate in patients with the lesion (43%) compared to patients where the lesions was not reported (13%)[36].

### Number of preoperative dislocations

Meta-analyses were feasible for > 1 preoperative dislocations and > 5 preoperative dislocations. Four studies (473 shoulders) demonstrated a higher recurrence risk in patients with > 1 dislocations lesion compared to patients with one dislocation (RR = 1.74; *P* = 0.03; *I*^2^ = 0%; Fig. 6); six studies (567 shoulders) demonstrated no difference when comparing patients with > 5 dislocations compared to patients with ≤ 5 dislocations (RR = 1.07; n.s.; *I*^2^ = 59%; supplementary 4, Figure 15).

### Time from first-time dislocation to surgical treatment and number of anchors

Meta-analyses were feasible for surgical delay of > 6 months from first-time dislocation to surgery and using ≤ 2 anchors during surgery. Two studies (565 shoulders) demonstrated a higher recurrence risk in patients with > 6 months delay compared to patients that received surgery within 6 months (RR = 2.86; *P* < 0.0001; *I*^2^ = 0%; supplementary 4, Figure 16); four studies (526 shoulders) demonstrated no difference when comparing patients with ≤ 2 anchors to patients with > 2 anchors (RR = 1.57; n.s.; *I*^2^ = 25%; supplementary 4, Figure 17).

### ISIS and MSTS

Meta-analyses were feasible for both ISIS > 3 and ISIS > 6. Seven studies (1,380 shoulders demonstrated a higher recurrence risk in patients with an ISIS > 3 compared to patients with an ISIS ≤ 3 (RR = 3.28; *P* = 0.0007; *I*^2^ = 77%; supplementary 4, Figure 18); four studies (1136 shoulders) demonstrated a higher recurrence risk in patients with an ISIS > 6 compared to patients with an ISIS ≤ 6 (RR = 4.88; *P* < 0.00001; *I*^2^ = 71%; supplementary 4, Figure 19). A meta-analysis for MSTS was not feasible, however, Kanatli et al. found a higher recurrence rate in patients with the MSTS (31%) compared to patients where the sign was not reported (7%)[23].

## Discussion

The most important findings of the present study were that age ≤ 20 years, age ≤ 30 years, participation in competitive sports, a Hill-Sachs lesion, an off-track Hill-Sachs lesion, glenoid bone loss, an ALPSA lesion, > 1 preoperative dislocations, > 6 months surgical delay from first-time dislocation to surgery, ISIS > 3 and ISIS > 6 were risk factors for recurrence following an arthroscopic Bankart repair. An off-track Hill-Sachs lesion and ISIS > 3 demonstrated the highest risk of recurrence with a RR > 3. Male gender, an affected dominant arm, hyperlaxity, participation in contact and/or overhead sports, a glenoid fracture, a SLAP lesion, a rotator cuff tear, > 5 preoperative dislocations and using ≤ 2 anchors could not be confirmed as risk factors for recurrence. In addition, no difference was observed between the age groups ≤ 20 years and 21–30 years.

This systematic review and meta-analysis includes several limitations. First, an I^2^ statistic of > 50% was observed in 11 meta-analyses [18]. This can be due to differences in study design, definitions or patient selection. For example, definition of hyperlaxity was not identical amongst studies, as different hyperlaxity tests were used. Second, this meta-analysis pooled averages and could not use individual patient data. This explains why the risk factors age ≤ 20 and ≤ 30 could only be analyzed separately and a multivariate analysis was not feasible. Third, most included studies had a retrospective design, inherently associated with missing data or biased outcomes. Fourth, this systematic review pooled the data of each risk factor, however the severity could not be included. Lesions vary in size and morphology, influencing recurrence risk [10]. Due to these limitations, the results should be interpreted with caution. However, this review includes strengths as well. A systematic approach was used and the search was constructed in cooperation with an information specialist. In addition, this is the first review that quantitatively evaluates these factors.

This review included the glenoid track concept and ISIS, which have both been validated in previous studies [2, 40, 41, 56]. The glenoid track concept takes both glenoid and humeral bone loss into account and may demonstrate when a bone augmentation procedure should be performed, as off-track lesions were confirmed as an important risk factor for recurrence following a Bankart repair. However, this cut-off value does not seem suitable to determine if a soft tissue repair is more beneficial than non-operative treatment for patients with small to no bone defects. A higher ISIS can be used to estimate recurrence risk, but the tool is probably too simplistic to determine a cut-off value that demonstrates when operative treatment is beneficial [6, 22, 35]. In addition, both type of sport and hyperlaxity are included in the ISIS and could not be confirmed as risk factors for recurrence in the quantitative analyses. Therefore, these items may need to be substituted by other factors or adjusted to be of additional value in this tool. For example, Nakagawa et al. demonstrated that the recurrence rate is significantly higher in rugby players compared to other collision or contact sports in competitive athletes [30]. This may indicate that contact and overhead sports should not be pooled and a more differentiated approach is needed for type of sport to be used in the ISIS. Moreover, SLAP lesions with/without repair and rotator cuff lesions were included in the analyses and could not be confirmed as risk factors. This in line with the recent meta-analysis by Feng et al. demonstrating that a combined SLAP and Bankart repair does not decrease recurrence rates compared to a Bankart repair alone [13]. The current meta-analysis for rotator cuff tears only included 19 cases and this sample size might have been too small to observe a difference. The incidence of rotator cuff lesions increases with age and it has been shown that older age is associated with lower recurrence rates [39, 50]. It is unclear if young patients with a rotator cuff tear demonstrate a higher risk for recurrence. As an accurate tool that can determine failure following treatment is lacking, the benefits and risks should be discussed with patients to make a shared decision.

Studies that determine risk factors for recurrence generally use dislocation or subluxation as objective failure following treatment or use a combination of objective and subjective failure as outcome [41]. Defining when treatment for anterior shoulder dislocations has failed can be challenging and an international consensus has yet to be reached. Park et al. have demonstrated that some factors, such as width of the Hill-Sachs lesion and number of preoperative dislocations, may increase the risk of patient dissatisfaction without recurrence following arthroscopic stabilization [34]. These factors did not necessarily match the factors that predicted objective failure. A different treatment strategy may have been more suitable for these patients even though they did not experience objective failure. Separately taking objective and subjective failure into account may both reduce social costs and increase patient satisfaction [9, 34, 47].

This meta-analysis pooled study data to identify risk factors and allow for more consensus regarding these factors. This can help professionals decide if a more invasive procedure (e.g. bone augmentation) is more beneficial for a patient to prevent recurrence compared to a labral repair. Future research should focus on clear definitions for risk factors and patient selection, preferably on an international scale. Prospective cohort study designs with a large sample size should be used to confirm if the factors identified in this review predispose recurrence following an arthroscopic Bankart repair. These studies should separately include subjective failure as well as objective failure to increase patient satisfaction and reduce social costs [9, 34, 47]. Currently, it is uncommon to publish anonymous individual patient data in orthopedic research. Sharing this data can be of additional value for pooling results in meta-analyses to increase samples sizes and homogeneity of the analyses [12]. In addition, this data can be used to create models that can determine recurrence risk for individual patients based on their profile [26].

## Conclusion

Meta-analyses demonstrated that age ≤ 20 years, age ≤ 30 years, participation in competitive sports, Hill-Sachs lesion, off-track Hill-Sachs lesion, glenoid bone loss, ALPSA lesion, > 1 preoperative dislocations, > 6 months surgical delay from first-time dislocation to surgery, ISIS > 3 and ISIS > 6 were risk factors for recurrence following an arthroscopic Bankart repair. These factors can assist clinicians in giving a proper advice regarding treatment.

## Supplementary Information

Below is the link to the electronic supplementary material.

Supplementary file1 (DOCX 17 KB)

Supplementary file2 (XLSX 51 KB)

Supplementary file3 (XLSX 16 KB)

Supplementary file4 (DOCX 213 KB)

## Abbreviations

ISIS: Instability severity index score
MINORS: Methodological index for non-randomized studies
ALPSA: Anterior labroligamentous periosteal sleeve avulsion
GLAD: Glenolabral articular disruption
SLAP: Superior labral anterior posterior
MSTS: Multiple subscapularis tendon sign
RR: Risk ratios
CI: Confidence interval
