# Supplementary material for: Age, participation in competitive sports, bony lesions, ALPSA lesions, > 1 preoperative dislocations, surgical delay and ISIS score > 3 are risk factors for recurrence following arthroscopic Bankart repair: a systematic review and meta-analysis of 4584 shoulders
Source: Knee Surg Sports Traumatol Arthrosc. 2021 Aug 22;29(12):4004–14. doi: 10.1007/s00167-021-06704-7 (PMC8595227; doi:10.1007/s00167-021-06704-7)
Supplement: Supplementary file 1 — Supplementary file1 (DOCX 17 KB) [file 167_2021_6704_MOESM1_ESM.docx]

**Supplementary files 1.** Full search strategy

All searches were conducted on November 12^th^ 2020. No filters or restrictions were applied.

*PubMed*

| #17 | Search:**#14 AND #15 AND #16**Sort by:**Most Recent** | 1,768 |
| --- | --- | --- |
| #16 | Search:**((("Recurrence"[Mesh] OR recurr*[tiab] OR relaps*[tiab] OR recrudesc*[tiab] OR repeat*[tiab]) AND ("Joint Dislocations"[Mesh] OR dislocat*[tiab] OR luxat*[tiab] OR instabilit*[tiab])) OR risk*[tiab] OR lesion*[tiab] OR (hill[tiab] AND sachs[tiab]) OR injur*[tiab] OR Perthes[tiab] OR ALPSA[tiab] OR (anterior[tiab] AND (labro[tiab] OR labral[tiab]) AND periosteal[tiab] AND sleeve[tiab] AND avulsion*[tiab]) OR HAGL[tiab] OR (humeral[tiab] AND avulsion*[tiab] AND glenohumeral[tiab] AND ligament*[tiab]) OR (greater[tiab] AND tuberosity[tiab]) OR fracture*[tiab] OR "Fractures, Bone"[Mesh] OR "Rotator Cuff"[Mesh] OR (rotator[tiab] AND cuff[tiab]) OR tear*[tiab] OR age[tiab] OR sport*[tiab] OR laxity[tiab] OR (glenoid[tiab] AND bone[tiab] AND loss[tiab]))**Sort by: **Most Recent** | 5,603,913 |
| #15 | Search:**(Bankart[tiab] OR "Bankart Lesions/surgery"[Mesh] OR arthroscopic stabilization[tiab] OR arthroscopic stabilisation[tiab] OR labral repair[tiab])**Sort by:**Most Recent** | 2,300 |
| #14 | Search:**("Shoulder Dislocation"[Mesh] OR "Shoulder"[Mesh] OR "Shoulder Joint"[Mesh] OR shoulder*[tiab] OR glenohumeral[tiab])**Sort by:**Most Recent** | 82,527 |

*Embase/Ovid*

| 1 | exp shoulder dislocation/ | 6512 |
| --- | --- | --- |
| 2 | exp shoulder/ | 83055 |
| 3 | (shoulder* or glenohumeral).ti,ab,kw. | 101743 |
| 4 | 1 or 2 or 3 | 138684 |
| 5 | (Bankart or arthroscopic stabilization or arthroscopic stabilisation or labral repair).ti,ab,kw. | 2813 |
| 6 | Bankart lesion/su [Surgery] | 198 |
| 7 | 5 or 6 | 2862 |
| 8 | (recurr* or relaps* or recrudesc* or repeat*).ti,ab,kw. | 1930525 |
| 9 | exp joint dislocation/ | 4059 |
| 10 | (dislocat*or luxat* or instabilit*).ti,ab,kw. | 154727 |
| 11 | 9 or 10 | 158430 |
| 12 | 8 and 11 | 19548 |
| 13 | (risk* or lesion* or (hill and sachs) or injur* or Perthes or ALPSA or (anterior and (labro or labral) and periosteal and sleeve and avulsion*) or HAGL or (humeral and avulsion* and glenohumeral and ligament*) or (greater and tuberosity) or fracture* or (rotator and cuff) or tear* or age or sport* or laxity or (glenoid and bone and loss)).ti,ab,kw. | 8234845 |
| 14 | exp fracture/ | 336756 |
| 15 | exp rotator cuff/ | 8999 |
| 16 | 12 or 13 or 14 or 15 | 8303779 |
| 17 | 4 and 7 and 16 | 2119 |

*Cochrane Database of Systematic Reviews & Cochrane Central Register of Controlled Trials*

| #1 | MeSH descriptor: [Shoulder Dislocation] explode all trees | 143 |
| --- | --- | --- |
| #2 | MeSH descriptor: [Shoulder] explode all trees | 537 |
| #3 | MeSH descriptor: [Shoulder Joint] explode all trees | 745 |
| #4 | (shoulder* or glenohumeral):ti,ab,kw | 11763 |
| #5 | #1 OR #2 OR #3 OR #4 | 11763 |
| #6 | MeSH descriptor: [Bankart Lesions] explode all trees and with qualifier(s): [surgery - SU] | 3 |
| #7 | (Bankart OR arthroscopic stabilization OR arthroscopic stabilisation OR labral repair):ti,ab,kw | 238 |
| #8 | #6 OR #7 | 238 |
| #9 | MeSH descriptor: [Recurrence] explode all trees | 12084 |
| #10 | (recurr* or relaps* or recrudesc* or repeat*):ti,ab,kw | 159845 |
| #11 | #9 OR #10 | 159894 |
| #12 | MeSH descriptor: [Joint Dislocations] explode all trees | 687 |
| #13 | (dislocat*or luxat* or instabilit*):ti,ab,kw | 5839 |
| #14 | #12 OR #13 | 6413 |
| #15 | #11 AND #14 | 1018 |
| #16 | (risk* or lesion* or (hill and sachs) or injur* or Perthes or ALPSA or (anterior and (labro or labral) and periosteal and sleeve and avulsion*) or HAGL or (humeral and avulsion* and glenohumeral and ligament*) or (greater and tuberosity) or fracture* or (rotator and cuff) or tear* or age or sport* or laxity or (glenoid and bone and loss)):ti,ab,kw | 549185 |
| #17 | MeSH descriptor: [Fractures, Bone] explode all trees | 6053 |
| #18 | MeSH descriptor: [Rotator Cuff] explode all trees | 344 |
| #19 | #15 OR #16 OR #17 OR #18 | 549508 |
| #20 | #5 AND #8 AND #19 | 145 |

*CINAHL/Ebsco*

| S18 | S3 AND S6 AND S17 | 729 |
| --- | --- | --- |
| S17 | S13 OR S14 OR S15 OR S16 | 1,482,038 |
| S16 | (MH "Rotator Cuff+") | 3,063 |
| S15 | (MH "Fractures+") | 58,529 |
| S14 | ( TI (risk* OR lesion* OR (hill AND sachs) OR injur* OR Perthes OR ALPSA OR (anterior AND (labro OR labral) AND periosteal AND sleeve AND avulsion*) OR HAGL OR (humeral AND avulsion* AND glenohumeral AND ligament*) OR (greater AND tuberosity) OR fracture* OR (rotator AND cuff) OR tear* OR age OR sport* OR laxity OR (glenoid AND bone AND loss)) ) OR ( AB (risk* OR lesion* OR (hill AND sachs) OR injur* OR Perthes OR ALPSA OR (anterior AND (labro OR labral) AND periosteal AND sleeve AND avulsion*) OR HAGL OR (humeral AND avulsion* AND glenohumeral AND ligament*) OR (greater AND tuberosity) OR fracture* OR (rotator AND cuff) OR tear* OR age OR sport* OR laxity OR (glenoid AND bone AND loss)) ) | 1,469,860 |
| S13 | S9 AND S12 | 4,294 |
| S12 | S10 OR S11 | 33,871 |
| S11 | ( TI (dislocat* OR luxat* OR instabilit*) ) OR ( AB (dislocat* OR luxat* OR instabilit*) ) | 31,033 |
| S10 | (MH "Dislocations+") | 8,266 |
| S9 | S7 OR S8 | 231,945 |
| S8 | ( TI (recurr* OR relaps* OR recrudesc* OR repeat*) ) OR ( AB (recurr* OR relaps* OR recrudesc* OR repeat*) ) | 212,296 |
| S7 | (MH "Recurrence") | 48,901 |
| S6 | S4 OR S5 | 1,126 |
| S5 | ( TI (Bankart OR arthroscopic stabilization OR arthroscopic stabilisation OR labral repair) ) OR ( AB (Bankart OR arthroscopic stabilization OR arthroscopic stabilisation OR labral repair) ) | 1,123 |
| S4 | (MH "Bankart Lesions/SU") | 58 |
| S3 | S1 OR S2 | 30,919 |
| S2 | ( Ti (shoulder* OR glenohumeral) ) OR ( AB (shoulder* OR glenohumeral) ) | 28,334 |
| S1 | (MH "Shoulder") OR (MH "Shoulder Dislocation") OR (MH "Shoulder Joint+") | 12,823 |

*Web of Science/Clarative*

TOPIC: (shoulder* OR glenohumeral) AND (Bankart or arthroscopic stabilization or arthroscopic stabilisation or labral repair) AND (((recurr* or relaps* or recrudesc* or repeat*) AND (dislocat*or luxat* or instabilit*)) OR risk* or lesion* or (hill and sachs) or injur* or Perthes or ALPSA or (anterior and (labro or labral) and periosteal and sleeve and avulsion*) or HAGL or (humeral and avulsion* and glenohumeral and ligament*) or (greater and tuberosity) or fracture* or (rotator and cuff) or tear* or age or sport* or laxity or (glenoid and bone and loss))

| **Database** | **Before deduplication** | **After deduplication** |
| --- | --- | --- |
| PubMed | 1768 | 1762 |
| Embase | 2119 | 580 |
| Cochrane Database of Systematic Reviews | 1 | 0 |
| Cochrane Central Register of Controlled Trials | 143 | 51 |
| CINAHL | 729 | 55 |
| Web of science | 2578 | 1136 |
| **Total** | 7338 | 3584 |
