# Supplementary material for: Age, participation in competitive sports, bony lesions, ALPSA lesions, > 1 preoperative dislocations, surgical delay and ISIS score > 3 are risk factors for recurrence following arthroscopic Bankart repair: a systematic review and meta-analysis of 4584 shoulders
Source: Knee Surg Sports Traumatol Arthrosc. 2021 Aug 22;29(12):4004–14. doi: 10.1007/s00167-021-06704-7 (PMC8595227; doi:10.1007/s00167-021-06704-7)
Supplement: Supplementary file 4 — Supplementary file4 (DOCX 213 KB) [file 167_2021_6704_MOESM4_ESM.docx]

**
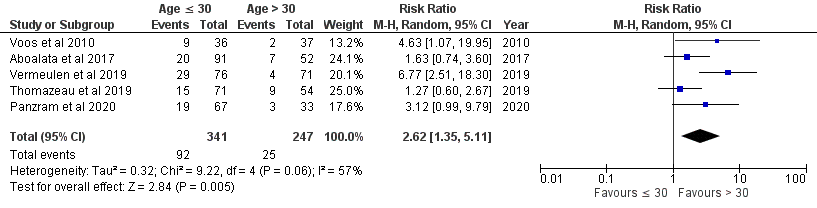
Figure 1 Meta-analysis of risk factor age ≤ 30 years.**

**
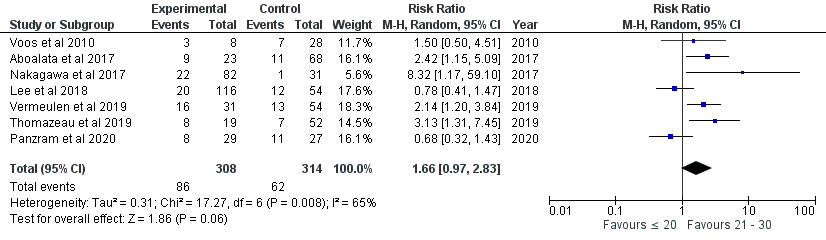
Figure 2 Meta-analysis of risk factor age ≤ 20 years versus 21-30 years.**

**
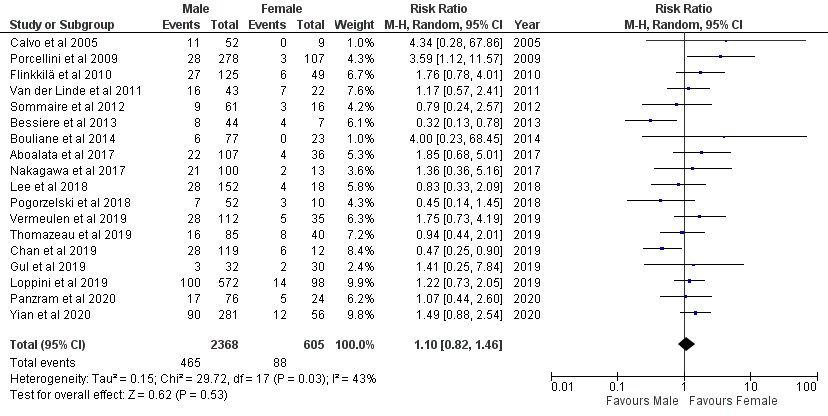
Figure 3 Meta-analysis of risk factor male gender.**

**
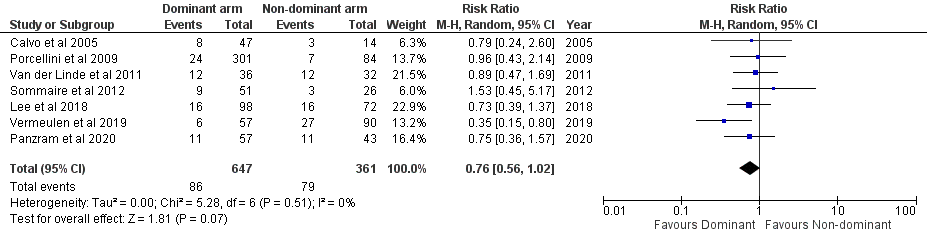
Figure 4 Meta-analysis of risk factor dominant arm affected.**

**
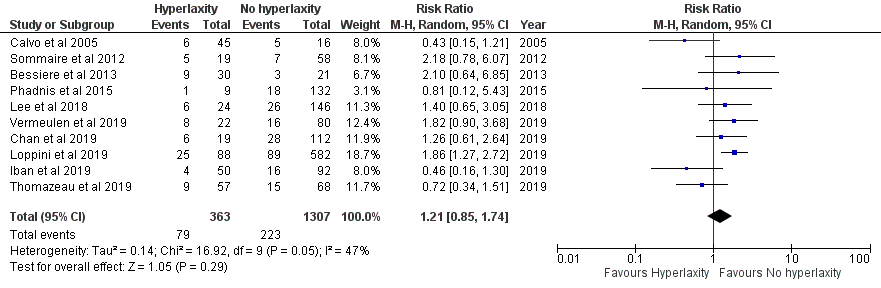
Figure 5 Meta-analysis of risk factor hyperlaxity**

**
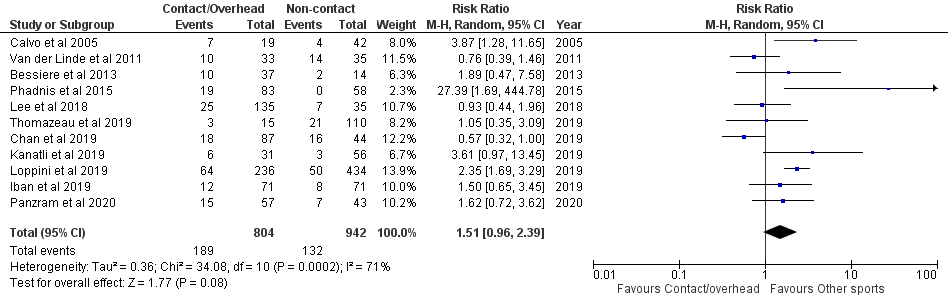
Figure 6 Meta-analysis of risk factor contact or overhead sports**

**
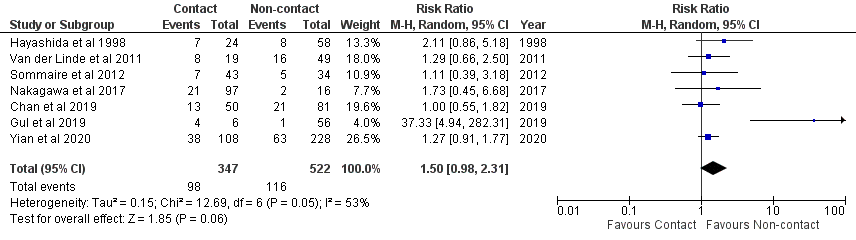
Figure 7 Meta-analysis of risk factor contact sports only**

**
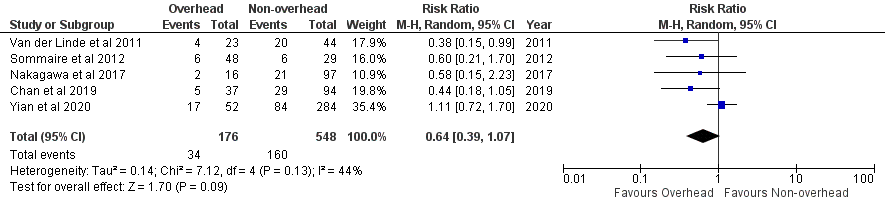
Figure 8 Meta-analysis of risk factor overhead sports only**

**Figure 9 Meta-analysis of risk factor off-track lesion**

**
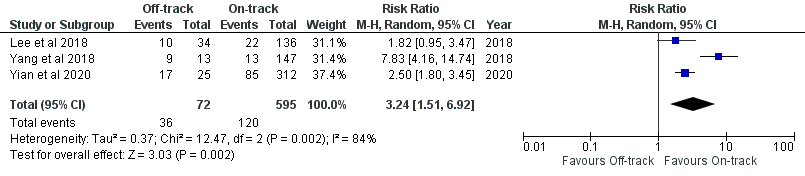
**

**
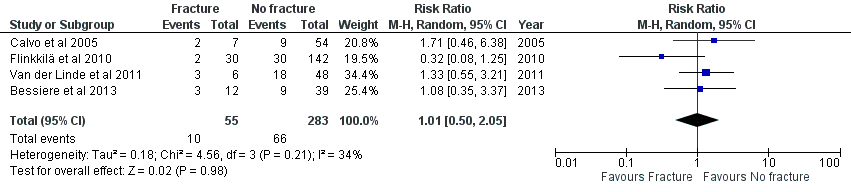
Figure 10 Meta-analysis of risk factor glenoid fracture**

**
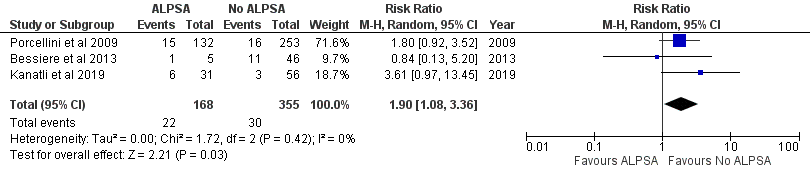
Figure 11 Meta-analysis of risk factor ALPSA lesion**

**
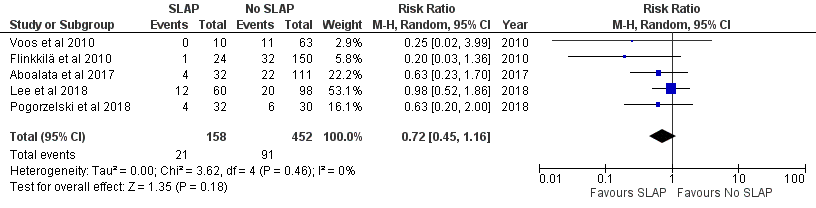
Figure 12 Meta-analysis of risk factor any SLAP lesion**

**
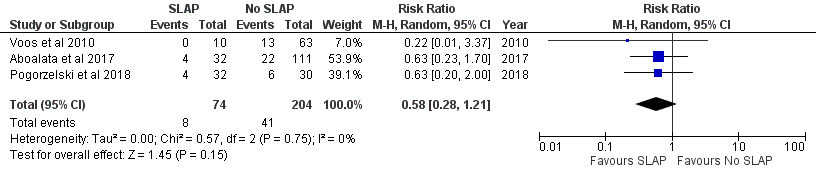
Figure 13 Meta-analysis of risk factor SLAP lesion with repair**

**
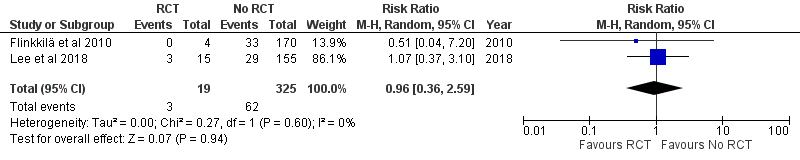
Figure 14 Meta-analysis of risk factor rotator cuff tear**

**
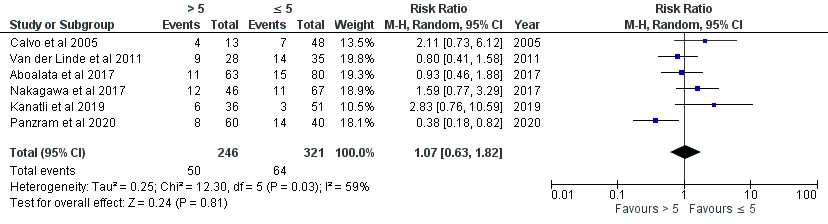
Figure 15 Meta-analysis of risk factor > 5 preoperative dislocations**

**
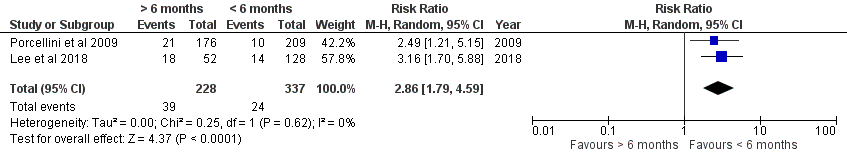
Figure 16 Meta-analysis of risk factor > 6 months delay**

**
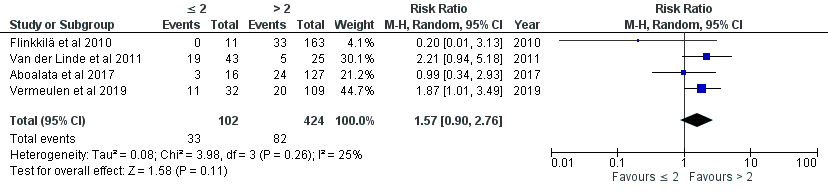
Figure 17 Meta-analysis of risk factor ≤ 2 anchors**

**
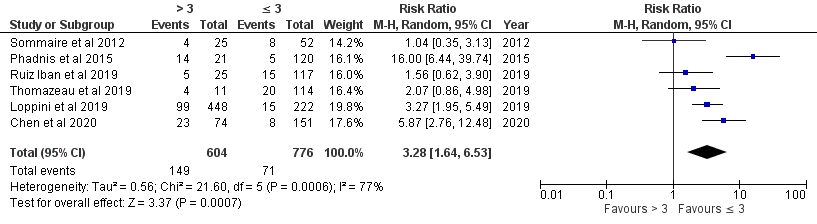
Figure 18 Meta-analysis of risk factor ISIS > 3**

**
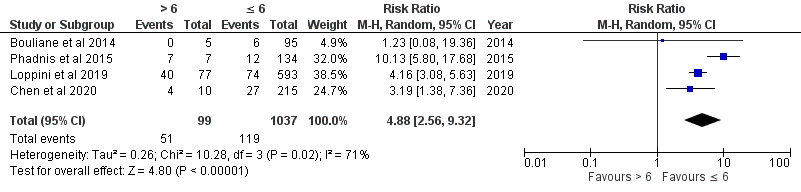
Figure 19 Meta-analysis of risk factor ISIS > 6**
